# Prevalence of mood and anxiety disorder in self reported irritable bowel syndrome (IBS). An epidemiological population based study of women

**DOI:** 10.1186/1471-230X-10-88

**Published:** 2010-08-05

**Authors:** Arnstein Mykletun, Felice Jacka, Lana Williams, Julie Pasco, Margaret Henry, Geoffrey C Nicholson, Mark A Kotowicz, Michael Berk

**Affiliations:** 1 Norwegian Institute of Public Health, Division of Mental Heath, Oslo, Norway; 2 University of Bergen, Faculty of Psychology, Section Mental Health Epidemiology, Norway; 3 The University of Melbourne, Department of Clinical and Biomedical Sciences: Barwon Health, Geelong, Australia; 4 Orygen Research Centre, Parkville, Australia; 5 Mental Health Research Institute, Parkville, Australia

## Abstract

**Background:**

Irritable bowel syndrome (IBS) is commonly regarded as a functional disorder, and is hypothesized to be associated with anxiety and depression. This evidence mainly rests on population-based studies utilising self-report screening instruments for psychopathology. Other studies applying structured clinical interviews are generally based on small clinical samples, which are vulnerable to biases. The extant evidence base for an association between IBS and psychopathology is hence not conclusive. The aim of this study was therefore to re-examine the hypothesis using population-based data and psychiatric morbidity established with a structured clinical interview.

**Methods:**

Data were derived from a population-based epidemiological study (n = 1077). Anxiety and mood disorders were established using the Structured Clinical Interview for DSM-IV-TR (SCID-I/NP) and the General Health Questionnaire (GHQ-12). Current and lifetime IBS was self-reported. Hypertension and diabetes were employed as comparison groups as they are expected to be unrelated to mental health.

**Results:**

Current IBS (n = 69, 6.4%) was associated with an increased likelihood of current mood and/or anxiety disorders (OR = 2.62, 95%CI 1.49 - 4.60). Half the population reporting a lifetime IBS diagnosis also had a lifetime mood or anxiety disorder. Exploratory analyses demonstrated an increased prevalence of IBS across most common anxiety and mood disorders, the exception being bipolar disorder. The association with IBS and symptoms load (GHQ-12) followed a curved dose response pattern. In contrast, hypertension and diabetes were consistently unrelated to psychiatric morbidity.

**Conclusions:**

IBS is significantly associated with anxiety and mood disorders. This study provides indicative evidence for IBS as a disorder with a psychosomatic aspect.

## Background

Gastrointestinal symptoms like heartburn, constipation, diarrhoea and nausea are common in the general population. In a large population-based health survey, 48% of participants reported at least one symptom during the last year [1]. Irritable Bowel Syndrome (IBS) is one of a number of Functional Bowel Disorders (FBDs) defined as "a functional bowel disorder in which abdominal pain or discomfort is associated with defecation or a change in bowel habit, and with features of disordered defecation" [2]. The prevalence of IBS is estimated to be in the range of 2.9 to 11.4% with variations across contexts and diagnostic criteria [3-5]. The overall prevalence of IBS was 11.5% in a survey of 40,000 individuals in 8 European countries [6]. The syndrome is reported to have a higher prevalence in women [3].

The organic aetiology of gastrointestinal symptoms is often unclear, but these symptoms are associated with symptoms of anxiety and depression [7] in a similar pattern to that found for other somatic symptoms that also have an uncertain organic aetiology [7,8]. Both clinical and population-based studies applying screening instruments for anxiety and depression have found similar increased rates of psychological distress in patients with IBS [4,9]. It has, however, been suggested that psychopathology among IBS patients is a function of other patient characteristics than the disease [10]. Anxiety and depression have been reported to independently increase health care utilization in patients with IBS [9,11], whereas a study utilising the General Health Questionnaire (GHQ-12) reported that IBS was associated with more psychiatric distress, regardless of medical care-seeking [12]. However, another well powered population-based study of young adults from New Zealand reported no association between IBS and symptoms of mental disorder [13]. Currently, there is no evidence for a common genetic component in the co-occurrence of Major Depressive Disorder (MDD) and IBS [14].

Little is known about factors influencing the onset of IBS. One study suggested poor quality of life as a risk factor [15] and another reported a history of abuse to be increased among subjects with IBS [16]. Others have suggested IBS to be a functional disorder, characterized by medically unexplained symptoms and often increased impairment [17-19]. The aetiology of functional disorders is still largely unknown, but it is believed that psychological stress may be a causative factor [20], and psychiatric comorbidities are common across functional disorders [18].

Despite many studies on psychiatric morbidity in IBS, there are still four issues to be addressed: First, the majority of studies are based on clinical samples rather than population samples. These studies are vulnerable to bias, as psychological morbidity is shown to increase help seeking in IBS [11], which may inflate observed associations and lead to type 1 errors. Second, relatively fewer studies have investigated the hypothesized association between psychiatric morbidity and IBS in community samples. Population-based studies need to be large as the prevalence of IBS is moderate [3-5]. Consequently, population-based epidemiological studies of the IBS-mental health association generally have to rely on self-reported symptoms of psychological distress rather than psychiatric diagnoses derived from thorough and expensive structured clinical interviews. To the best of our knowledge, no population-based studies exist examining the IBS-mental health association utilising psychiatric diagnoses derived from clinical interviews. And third, not all studies of the IBS-mental health association have reported positive findings [13]. Among clinical studies using the SCID interview, there are also negative findings [21]. And fourth, not only are many of the reported positive associations based on potentially biased clinical samples, but also on relatively small sample sizes. There is also potential publication bias in favour of positive findings.

The aim of the present study was to re-examine the hypothesized IBS-mental disorder association in the context of a relatively large (n = 1077) population-based health study, applying interview based clinical diagnoses for psychiatric morbidity in addition to self-administered screening instruments. To exclude the possibility of bias producing observed associations between psychopathology and IBS, we also examined the associations of diabetes and hypertension to psychopathology. These comparison disorders were chosen as they are not regarded to be functional disorders, and we expected weak or no associations of these disorders with psychopathology [22-24].

## Methods

### Study design

The Geelong Osteoporosis Study (GOS) is a large epidemiological study involving subjects selected from electoral rolls for the Barwon Statistical Division, a region defined by the Australian Bureau of Statistics. The area is well suited to epidemiological research as it has a defined population consisting of a range of social, cultural and geographical settings, with a centralised health provider. The total population and female population according to the 2006 census was 259,013 and 132,124 (98,740 aged 20 years and over), respectively. An age-stratified, random, population-based sample of 1,494 women (ages 20-94 yr) was recruited between 1994 and 1997 with a minimum of 100 in each 5-yr age stratum between ages 20 and 69 and 200 in the age 70-79 yr group and in the over 80 yr group. Of the 2,390 eligible invitees, 1,494 women consented to participate, with a response of 77.1% [25]. These women have continued to return for biennial assessment with participation rates of between 70-88%. A further cohort of 200 women aged 20-29 years was also recruited at the time of the 10-yr follow up to allow for the full adult age range to continue to be investigated. Comparisons between the Barwon Statistical Division characteristics and the Australian female population indicate that this is a representative sample of Australian women [25].

From a potential pool of 1094 women who had participated in the GOS 10-year follow-up, participants for whom psychiatric or medical data were not available (n = 17) were excluded from the analyses, resulting in a final sample of 1077 women aged 20-93 years available for analysis. All participants were female, and the age distribution was almost uniform as a consequence of the sampling procedure, with sample sizes across decades ranging from 20-29 to 80-89 of n = 201, 147, 175, 179, 160, 134 and 81 respectively. The Barwon Health Human Research Ethics Committee approved the study, and written informed consent was obtained from all participants.

### Assessment of psychiatric conditions by personal interviews

Two specially trained interviewers (LW and FJ, co-authors of this paper) performed all interviews personally. The Structured Clinical Interview for DSM-IV-TR Research Version, Non-patient edition (SCID-I/NP) was used. This is a validated, semi-structured clinical interview for the major axis I psychiatric disorders in the DSM-IV-TR. Trained researchers, blinded to the participant's medical information, carried out the SCID-I/NP assessments. Psychiatric disorders assessed included major depressive disorder, minor depression, dysthymia, and bipolar disorder. Past and current anxiety disorders including panic disorder, agoraphobia, social phobia, specific phobia, obsessive-compulsive disorder, generalised anxiety disorder (current only), anxiety disorders due to a general medical condition, substance induced anxiety disorder and anxiety disorders not otherwise specified were also assessed.

The General Health Questionnaire (GHQ-12) was used as an additional continuous measure of depressive symptoms [26]. This is a well-established screening instrument designed to detect non-psychotic psychiatric disorders in community and medical settings using a 12-item self-report questionnaire based on the respondent's assessment of their present state. Each item is scored on a four-point Likert scale. To create a continuous score with as wide a range as possible, these answers were summed to give a final score for each participant with a potential range of 12-48.

A GHQ-12 total scale cut-off at the 80^th ^percentile was defined for categorical analyses, and was used for secondary analyses. The Kappa between the 80^th ^percentile GHQ-12 (defining 218 cases among 1060 with valid responses to the GHQ-12) and current anxiety or mood disorder as defined by the SCID interview was 0.35 (84 overlapping cases among 147 individuals with current SCID-defined psychiatric morbidity).

### Assessment of IBS

Current and lifetime IBS was assessed in a clinical interview conducted prior to the SCID-I/NP interview. Participants were asked whether or not they had ever had IBS, which was included as one of many medical conditions. In addition, participants were asked to state the age of onset and whether or not the condition was present in the last 12 months.

### Assessment of diabetes and hypertension

Hypertension was defined as either systolic or diastolic blood pressure above the 95^th ^percentile, or current medication for hypertension. Cut-offs for 95^th ^percentile blood pressure were based on age- and gender stratified population data [27]. Appropriately qualified research assistants measured blood pressure using a digital meter (A&D Company, model UA-751) with the cuff placed on the right upper arm and with the arm resting on a table and the participant seated.

Current diabetes was self-reported on the same basis as the IBS report. Self-report of diabetes is regarded to be reliable [27]. Additionally, data regarding medication use for both diabetes and hypertension was also self-reported, and these data were used to corroborate the self-reported medical conditions. Both hypertension and diabetes were chosen as comparison groups on the basis of well-established organic aetiology, and reports of weak or non-existing associations with psychiatric conditions [22-24].

### Confounders

Age and education were identified and tested as potential confounding variables. Information regarding the highest educational level attained was derived from baseline assessments, and comprised five categories: from completed primary school (1) through to university or other tertiary qualification (5). The educational level was widely distributed with n = 491 in level 1 and 2 and n = 300 in level 5. IBS (current and lifetime) were not significantly related to age and educational level (all p > .18). Diabetes and hypertension were associated with both age and educational level in directions as expected (p < .001). The associations of psychiatric conditions with educational level and age were not significant (all p > .071), however, lifetime psychiatric conditions, and any lifetime anxiety disorder in particular, were negatively associated with age (p < .001 and p = .017 respectively). Nutritional factors are well covered in this sample, and we have previously found unhealthy eating habits to be associated with mental disorders [28]. However, nutrition was excluded as a potential mechanism for the association of interest as it was unrelated to IBS (all p > .505). On the basis of these bivariate associations, age and educational level were included as potential confounding factors in further analyses. Further adjustment for measured BMI and waist circumference was examined, but omitted from analyses, as they did not account for the associations of interest.

### Analysis and statistics

First, we cross-tabulated current IBS, diabetes and hypertension across current mood and anxiety disorders. Observed numbers and percentages of psychiatric conditions within IBS, diabetes and hypertension were estimated. Using the Chi-Square test and Fisher Exact test in cases with expected counts in any cell of five or less, the significance of associations was tested. These analyses were then repeated for lifetime IBS versus lifetime mood or anxiety disorders.

Logistic regression analyses were employed to estimate the associations between IBS, diabetes and hypertension (dependent variables) and psychiatric morbidity. Models were adjusted for age and educational level. As these adjustments had only marginal influence on observed associations, adjusted models only were reported in tables.

In order to compare the results with studies applying self-report screening instruments for psychopathology, all models were repeated applying the 80^th ^percentile on the GHQ-12 as the exposure variable. Additionally, the percentage of those reporting IBS was examined in each centile of GHQ-12 score. The dose response association was examined with regression models with IBS as dependent variable, and GHQ (centiles) as independent. Two extra models were included: First, we excluded the two last centiles of the GHQ distribution, examining if the eventual response association was apparent also within the sub-clinical distribution. Second, we examined eventual curve linearity in the IBS GHQ association by including also a quadratic term (GHQ centile variable squared).

Finally, exploratory analyses examining IBS across all psychiatric diagnoses (both current and lifetime) were performed. We had no *a priori *hypotheses regarding which psychiatric conditions were expected to be associated with IBS. Results were presented as cross tabulations, and as odds ratios with 95% confidence intervals, adjusted for age and educational level.

## Results

Current IBS was reported in 6.4% (69 of 1077 study participants; 95% CI 4.9 - 7.9). The prevalence of lifetime IBS was 8.4% (95% CI 6.8 - 10.1). No associations between IBS and age or educational level were found. The prevalence of IBS was comparable to the prevalence of diabetes (5.5%, 95% CI 4.1 - 6.8). Hypertension was defined in 30.9% of participants (95% CI 28.2 - 33.7).

IBS was consistently associated with mood and anxiety disorders. Among participants reporting current IBS, 27.5% (95% CI 17.0 - 38.1) also fulfilled the criteria for a current anxiety or mood disorder (table 1). In comparison, the prevalence of a current anxiety or mood disorder was 14.0% (95% CI 11.9 - 16.1) across all study participants. After adjustment for age and educational level, the odds ratio for current anxiety or mood disorder among IBS sufferers was 2.62 (95% CI 1.49 - 4.60) (Table 1). The association between current IBS with current mood disorder was comparable to that of the current anxiety disorder. Lifetime IBS and lifetime mood or anxiety disorder followed the same pattern. Among 91 lifetime IBS cases, 46 (50.5%, 95% CI 40.3 - 60.8) had a lifetime diagnosis of mood or anxiety disorder, equivalent to an adjusted odds ratio of 2.12 (95% CI 1.37 - 3.29) (Table 2).

**Table 1:** Current IBS, hypertension and diabetes versus current mood disorder, anxiety disorder, and GHQ 80^th ^percentile case-level.

		Current mood or anxiety disorder	Current mood disorder	Current anxiety disorder	**GHQ >= 80**^**th **^**percentile #**
Total sample		151 [14.0%] (11.9 - 16.1)	91 [8.4%] (6.8 - 10.1)	83 [7.7%] (6.1 - 9.3)	217 [20.6%] (17.8 - 22.5)
Current IBS	69	19 [27.5%]** (17.0 - 38.1)	11 [15.9%]* (7.3 - 24.6)	13 [18.8%]** (9.6 - 28.1)	31 [44.9%]** (33.2 - 56.7)
Diabetes	59	8 [13.6%] (4.8 - 22.3)	5 [8.5%] (1.4 - 15.6)	5 [8.5%] (1.4 - 15.6)	10 [17.1%] (7.4 - 26.5)
Hypertension	333	48 [14.4%] (10.6 - 18.2)	31 [9.3%] (6.2 - 12.4)	25 [7.5%] (4.7 - 10.3)	61 [19.0%] (14.2 - 22.5)

Numbers are observed N among total N = 1077 and [% for rows] and (95% CI)

* p < .001, ** p < .05, # Missing data in 17 individuals. Other data complete.

**Table 2:** Lifetime IBS versus lifetime mood disorder, anxiety disorder, and GHQ 80^th ^percentile case-level.

	Lifetime mood or anxiety disorder	Lifetime mood disorder	Lifetime anxiety disorder	**GHQ >= 80**^**th **^**percentile #**
Total sample	366 (34.0%)	308 (28.6%)	143 (13.3%)	207 (20.6%)
Lifetime IBS (N = 91)	46 (50.5%)**	40 (44.0%)**	19 (20.9%)*	40 (44.0%)**
Adjusted odds ratio (95% CI)	2.12 (1.37 - 3.29)	2.08 (1.34 - 3.24)	1.86 (1.08 - 3.21)	3.48 (2.23 - 6.42)

Numbers are observed N among total N = 1077 and (% for rows).

* p < .001, ** p < .05, # Missing data in 17 individuals. Other data complete.

No associations between mood or anxiety disorders and diabetes or hypertension were found, either in unadjusted (table 1) or adjusted models (table 3).

**Table 3:** Current psychiatric disorders (independent variables) in relation to current IBS, diabetes, and hypertension (dependent variables), adjusted for age and educational level.

	Current mood or anxiety disorder	Current mood disorder	Current anxiety disorder	GHQ >= 80th percentile #
Current IBS	2.62 (1.49 - 4.60)	2.29 (1.15 - 4.56)	3.19 (1.65 - 6.17)	3.69 (2.23 - 6.12)
Diabetes	1.14 (0.52 - 2.53)	1.18 (0.45 - 3.11)	1.41 (0.53 - 3.77)	0.77 (0.38 - 1.58)
Hypertension	1.26 (0.84 - 1.89)	1.39 (0.85 - 2.27)	1.17 (0.69 - 2.00)	0.85 (0.59 - 1.22)

Odds ratios with 95% confidence interval.

# Missing data in 17 individuals. Other data complete.

Secondary analyses of the same associations, applying the 80^th ^percentile cut-off of the GHQ-12 as marker of psychiatric morbidity, confirmed all findings (table 1, 2 and 3). Associations tended to be somewhat stronger applying the GHQ-12 cut-off for 80^th ^percentile than for analyses based on SCID-I/NP-diagnosed psychiatric conditions.

**Figure 1:**
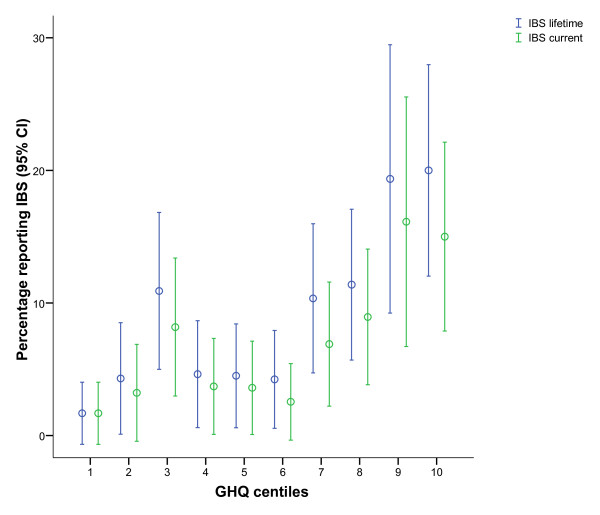
**Dose response association between current symptom load of anxiety and depression (GHQ-12), and percent reporting IBS (tests for trend p < .001)**. Dose response association for whole range (p < .001), and also excluding the two last centiles of the GHQ distribution (p = .040 and p = .008 for current and lifetime respectively). The apparent curve-linearity in the GHQ IBS association was statistically significant (p = .021 and p = .019 for current and lifetime respectively).

A dose-response association was observed between symptoms of anxiety and depression (as defined by GHQ-12 centiles) and prevalence of lifetime and current IBS (figure 1). Pearson correlations between GHQ centiles and IBS was 0.14 and 0.17 for current and lifetime IBS respectively (p < .001). The dose response association was statistically significant also within the sub-clinical range of the GHQ distribution (excluding the two last centiles) with person correlations of 0.07 (p = .040) and 0.09 (p = .008) for current and lifetime respectively. There was a significant curve linearity in the IBS GHQ association (p-values for quadratic terms for GHQ 0.021 and 0.019 for current and lifetime, respectively).

Finally, we performed exploratory analyses of psychiatric diagnoses across IBS. With few exceptions, all psychiatric conditions were increased in IBS. Among 10 current psychiatric conditions with 10 or more cases (prevalence of approximately 1% or more), positive associations were observed for all except bipolar spectrum disorders. However, only two of these associations (minor depression and generalized anxiety disorder) were statistically significant (table 4). A similar pattern was found for lifetime psychiatric conditions: among 12 lifetime psychiatric conditions with a prevalence of 1% or more, positive associations with IBS were observed for 11 of these. However, only lifetime major depressive disorder (MDD), MDD with recurrent episodes, and minor depression were statistically significant (table 5). Examining the largely overlapping confidence intervals, there was no solid empirical basis to conclude an association between IBS and any specific psychiatric condition (table 4 and 5).

**Table 4:** Specific current psychiatric diagnoses in relation to current and lifetime IBS

				Current IBS					Lifetime IBS				
				
		Total prevalence	% of total	With IBS	% with IBS	Fisher exact test	OR	95% CI	With IBS	% with IBS	Fisher exact test	OR	95% CI
MDD	+	49	4.5%	5	7.2%	.229	1.9	0.7 - 4.9	5	5.5%	.591	1.3	0.5 - 3.5
MDD Dysthmia	+	63	5.8%	6	8.7%	.280	1.7	0.7 - 4.1	6	6.6%	.639	1.2	0.5 - 2.9
Dysthymia	+	19	1.7%	2	2.9%	.339	1.7	0.4 - 7.7	2	2.2%	.669	1.3	0.3 - 5.6
Bipolar Spectrum	-	24	2.2%	1	1.4%	1.000	0.6	0.1 - 4.8	2	2.2%	1.000	0.9	0.2 - 4.1
Minor Depression*	+	13	1.2%	4	5.8%	.007	6.8	2.0 - 23.0	4	4.4%	.018	5.1	1.5 - 17.0
Generalised Anxiety Disorder*	+	10	0.9%	4	5.8%	.002	10.9	2.9 - 40.2	4	4.4%	.007	7.5	2.1 - 27.5
Panic Disorder	+	14	1.3%	2	2.9%	.219	2.5	0.6 - 11.8	2	2.2%	.331	1.8	0.4 - 8.5
Agoraphobia with no panic disorder	-	6	0.5%	0	0.0%				0	0.0%			
Social Phobia	+	10	0.9%	2	2.9%	.127	3.8	0.8 - 18.3	2	2.2%	.202	2.7	0.6 - 12.9
Specific Phobia	+	32	2.9%	4	5.8%	.137	2.2	0.7 - 6.6	5	5.5%	.181	2.1	0.8 - 5.8
Obsessive Compulsive Disorder*	+	6	0.5%	2	2.9%	.050	10.1	1.6 - 61.9	2	2.2%	.084	7.2	1.2 - 43.8
Anxiety, not otherwise specified	+	12	1.1%	1	1.4%	.544	1.3	0.2 - 10.7	1	1.1%	1.000	1.0	0.1 - 7.8

Psychiatric conditions with a prevalence of n < 6 were excluded for power reasons.

* p < .05 for current or lifetime IBS

+ association (significant or not) in positive direction

- association (significant or not) in negative direction

**Table 5:** Specific lifetime psychiatric diagnoses in relation to current and lifetime IBS

				Current IBS					Lifetime IBS				
				
		Total prevalence	% of total	With IBS	% with IBS	Fisher exact test	OR	95% CI	With IBS	% with IBS	Fisher exact test	OR	95% CI
MDD*	+	247	22.6%	24	34.8%	.017	2.0	1.2 - 3.4	30	33.0%	.018	1.8	1.1 - 2.9
MDD Single Episode	+	121	11.1%	8	11.6%	.843	1.1	0.5 - 2.3	13	14.3%	.290	1.4	0.7 - 2.6
MDD Recurrent Episodes*	+	126	11.5%	16	23.2%	.005	2.7	1.5 - 4.9	17	18.7%	.039	2.0	1.1 - 3.4
MDD Dysthmia	+	63	5.8%	6	8.7%	.280	1.7	0.7 - 4.1	6	6.6%	.639	1.2	0.5 - 2.9
Bipolar Spectrum	-	24	2.2%	1	1.4%	1.000	0.6	0.1 - 4.8	2	2.2%	1.000	0.9	0.2 - 4.1
Minor Depression*	+	31	2.8%	4	5.8%	.126	2.2	0.7 - 6.4	6	6.6%	.039	2.6	1.0 - 6.6
Panic Disorder	+	59	5.4%	5	7.2%	.413	1.4	0.5 - 3.6	7	7.7%	.330	1.5	0.7 - 3.4
Agoraphobia with no panic disorder	-	8	0.7%	0	0.0%				0	0.0%			
Social Phobia	+	23	2.1%	2	2.9%	.652	1.3	0.3 - 5.9	2	2.2%	1.000	1.0	0.2 - 4.4
Specific Phobia	+	36	3.3%	4	5.8%	.279	1.9	0.6 - 5.6	5	5.5%	.218	1.8	0.7 - 4.8
Obsessive Compulsive Disorder	+	14	1.3%	2	2.9%	1.000	2.7	0.6 - 12.7	2	2.2%	1.000	1.9	0.4 - 8.9
Anxiety NOS	+	17	1.6%	2	2.9%	.292	2.0	0.4 - 9.0	2	2.2%	.647	1.4	0.3 - 6.4

Psychiatric conditions with a prevalence of n < 6 were excluded for power reasons.

* p < .05 for current or lifetime IBS

+ association (significant or not) in positive direction

- association (significant or not) in negative direction

## Discussion

### Main findings

IBS was consistently associated with psychopathology in this study. Among individuals with current IBS, 27.5% also had a current psychiatric mood or anxiety disorder (OR = 2.62), and among lifetime IBS, 50.5% also had a lifetime psychiatric condition (OR = 2.12). Associations of IBS to psychopathology were equally strong in mood and anxiety disorders, and the prevalence of all psychiatric conditions except bipolar disorder tended to be increased in IBS (though not all statistically significant).

### Strengths and limitations

There are three main strengths of this study, which makes it a novel contribution to the existing literature: Psychiatric conditions were defined applying semi-structured clinical interviews rather than self-report by screening instruments only. This increased the reliability and validity of psychopathology diagnoses, and enabled us to examine IBS across psychiatric diagnoses. The findings reflected in the categorical data were supported by the GHQ-12 dimensional measures of psychopathology. The study sample was population-based, avoiding potential selection biases emerging from increased help seeking on the basis of psychopathology in IBS [11], which might inflate effect sizes, and potentially produce type I errors. Finally, the study was generously powered to examine the association of IBS to general psychopathology.

The main limitation of this study is that the IBS diagnosis was based on self-report rather than the use of one or more of the established diagnostic criteria (Rome I, Rome II or Manning). IBS may represent heterogeneous conditions, and our crude measure of IBS obstructed analyses of eventual variations in occurrence of mental disorders across IBS conditions. We cannot exclude there being several different causal paths for different IBS conditions. In the large European study (overall prevalence 11.5%, n = 41,984) only 4.8% had been formally diagnosed but a further 4.2%, 2.9% and 6.5% met the Rome I, Rome II or Manning criteria respectively [6].

Other studies have also shown that different formal definitions of IBS result in very different prevalence rates for the disorder [3-5]. The prevalence of current IBS in our study (6.4%, 95% CI 4.9 - 7.9%) is between the prevalences applying the Rome II (2.9%) and III (11.4%) criteria [3]. In an Australian population-based study set in New South Wales (n = 762, 51.1% female, response rate 62.2%) 8.9% met Rome II criteria for IBS [29]. In a larger Australian cohort (n = 4500, response rate 72%), the prevalence for IBS according to Manning, Rome I, and Rome II criteria was 13.6%, 4.4%, and 6.9%, respectively [30]. The concordance with definitions according to formal criteria for IBS cannot be established based on our data. However, it is unlikely that poor validity of our measure of IBS would alone produce the observed associations between IBS and psychopathology.

Both IBS [3] and common mental disorder [31] are more common in women than men. Inclusion of women only in this study is thus relevant for the association of interest. While the GOS female cohort is known to be largely representative of the Australian female population [25], inclusion of women only obviously restricts the generalisability of our findings. Furthermore, it is known that non-participation in health studies is associated with increased prevalence of mental disorders [32], disability [33], and mortality [34]. It is thus likely our sample is biased in a healthy direction, which might deflate observed associations. Finally, by virtue of the cross-sectional design, causality cannot be inferred.

The relatively low concordance between 80^th ^percentile case-level on the GHQ-12 and current anxiety or mood disorder (kappa 0.35) indicates that the findings of studies applying screening instruments only partly describe the same cases as do studies applying thorough clinical interviews to establish psychiatric morbidity. Screening instruments like the GHQ-12 are generally regarded as rough and approximate indicators of psychopathology compared to the "gold standards" of clinical diagnoses. Given this, it is perhaps somewhat surprising that the associations of IBS to GHQ-12 defined case-level for the arbitrary 80^th ^percentile appeared to be stronger than to that of clinical diagnosis. However, this is consistent with previous findings where screening instruments have impressive predictive validity many years ahead for adverse outcomes like mortality and disability [35-37]. Further, examining confidence intervals, we cannot exclude the possibility of this being a chance finding, and the differences are by no means consistently statistically significant. One explanation for the difference might be that the broad group as defined by any current mood or anxiety disorder is very heterogeneous, including, for example, both the severe conditions of bipolar disorder and relatively more benign conditions such as specific phobias.

### Interpretation

Our finding of an IBS-mental health association is in line with previous reports of positive associations [4,9], but contrasts with some population-based studies [13] and studies applying clinical interviews for psychopathology [21]. Our finding of increased psychopathology in IBS is not new. The novelty of our study is mainly related to improved methodology: psychopathology defined by clinical interview; a population based design; and generous statistical power.

The most striking exception from the associations between IBS and various mental disorders was bipolar disorder. This might well be a type 2 error due to sample size. If replications confirm this lack of association, it should be investigated whether this lack of association is specific to IBS, or if is a pattern (or lack of such) across other conditions.

The lack of associations of psychopathology to diabetes and hypertension was as expected, and in accordance with findings from previous large population-based studies [22-24]. These negative findings might be of value for future meta-analyses. For the purpose of this study, the lack of association of diabetes and hypertension with psychopathology indicates that the observed associations for IBS are not only a product of response bias or selection bias.

How strong is the association of IBS and psychopathology? Despite consistent associations, the majority (72.5%) of individuals with current IBS had no current psychopathology as defined by the structured clinical interview. Applying the slightly more inclusive 80^th ^percentile on the GHQ-12 identified 55.1% without case-level GHQ-12 score among individuals with current IBS. Among lifetime IBS, 49.5% had no lifetime psychiatric diagnosis. Despite thorough clinical interviews for psychiatric morbidity, we cannot exclude false negative cases. Further, the contribution of sub-clinical case-levels cannot be excluded. However, there is no evidence, neither in this nor previous studies, for a history of psychopathology in *all *individuals with IBS. The opposite might also in some cases be true, that abdominal pain or IBS preceded the psychiatric condition.

Our finding of an association between lifetime psychiatric morbidity and current IBS is in accordance with studies demonstrating that somatisation is associated with incident IBS [38]. Others have found somatisation to play a key role in explaining IBS, but not dyspepsia [39]. Unfortunately, our study included no information on somatisation, but the correlation between symptoms of somatisation and symptoms of anxiety and depression is reported to be strong in the general population [40].

IBS is suggested to be a functional disorder [17-19]. The term functional somatic syndrome is applied to several related syndromes characterized more by symptoms, suffering, and disability than by consistently demonstrable organic or tissue abnormality. Other functional disorders include multiple chemical sensitivity; the sick building syndrome; repetitive stress injury; the side effects of silicone breast implants; the Gulf War syndrome; chronic whiplash; the chronic fatigue syndrome; and fibromyalgia [18]. Somatic symptoms are often referred to as medically unexplained or functional when the physician can find no objective organic explanation to the patient's symptoms or sufferings [17]. The aetiology of functional disorders is still largely unknown. The existence of many distinct functional somatic syndromes has been questioned, with reference to overlap in case definitions, reported symptoms, and non-symptom associations [17,19,41]. It has been suggested that specific functional disorders are mainly an artefact of medical specialisation [17]. The prevalence of specific functional disorders varies with geography and over time [17,18]. However, increased prevalence of psychiatric disorders, particularly anxiety and mood disorders, seem to be a common feature across functional somatic syndromes. Psychosocial stress and psychopathology are increased in all functional disorders, but not in all individuals with the functional disorders, and is generally thought of as being the cause rather than the consequence of functional disorders [18]. The consistent association of IBS with psychopathology is indicative, but not conclusive, evidence of IBS as a functional disorder.

## Conclusion

IBS is significantly associated with anxiety and mood disorders. This study provides indicative evidence for IBS as a disorder with a psychosomatic aspect.

## List of abbreviations used

IBS: Irritable Bowel Syndrome; MDD: Major Depressive Disorder; GHQ-12: General Health Questionnaire, 12 item version; SCID-I/NP: Structured Clinical Interview for DSM-IV-TR; GOS: Geelong Osteoporosis Study.

## Competing interests

None of the authors received any particular support for this study. The authors have no conflicts of interest. The funding providers played no role in the design or conduct of the study; collection, management, analysis, and interpretation of the data; or in preparation, review, or approval of the manuscript.

## Authors' contributions

AM: Study conception and design, analysis and interpretation of data, drafting of article, final approval of the version for publication. FJ& LW: Data collection, study design, interpretation of data, critical revision of manuscript for important intellectual content, final approval of the version for publication. MB & JP: Study design, critical revision of manuscript for important intellectual content, final approval of the version for publication. GN & MK: Conception of the GOS, critical revision of manuscript for important intellectual content, final approval of the version for publication.

## Pre-publication history

The pre-publication history for this paper can be accessed here:

http://www.biomedcentral.com/1471-230X/10/88/prepub
